# Transmission electron microscopy study of suspected primary ciliary dyskinesia patients

**DOI:** 10.1038/s41598-022-06370-w

**Published:** 2022-02-11

**Authors:** Mitra Rezaei, Amirali Soheili, Seyed Ali Ziai, Atefeh Fakharian, Hossein Toreyhi, Mihan Pourabdollah, Jahangir Ghorbani, Mahboobeh Karimi-Galougahi, Seyed Alireza Mahdaviani, Maryam Hasanzad, Alireza Eslaminejad, Hossein Ali Ghaffaripour, Saied Mahmoudian, Zahra Rodafshani, Maryam Sadat Mirenayat, Mohammad Varahram, Majid Marjani, Payam Tabarsi, Davood Mansouri, Hamid Reza Jamaati, Ali Akbar Velayati

**Affiliations:** 1 Department of Pathology, School of Medicine, Shahid Beheshti University of Medical Sciences, Tehran, Iran; 2 Clinical Tuberculosis and Epidemiology Research Centre, National Research Institute of Tuberculosis and Lung Diseases (NRITLD), Shahid Beheshti University of Medical Sciences, Tehran, Iran; 3 Medical Student Research Committee, School of Medicine, Shahid Beheshti University of Medical Sciences, Tehran, Iran; 4 Department of Pharmacology, School of Medicine, Shahid Beheshti University of Medical Sciences, Tehran, Iran; 5 Chronic Respiratory Diseases Research Center (CRDRC), National Research Institute of Tuberculosis and Lung Diseases (NRITLD), Shahid Beheshti University of Medical Sciences, Tehran, Iran; 6 Tracheal Diseases Research Center, National Research Institute of Tuberculosis and Lung Diseases, Shahid Beheshti University of Medical Sciences, Tehran, Iran; 7 Pediatric Respiratory Disease Research Centre, National Research Institute of Tuberculosis and Lung Diseases (NRITLD), Shahid Beheshti University of Medical Sciences, Tehran, Iran; 8 Central Lab, School of Medicine, Shahid Beheshti University of Medical Sciences, Tehran, Iran

**Keywords:** Cell biology, Diseases, Molecular medicine

## Abstract

Primary ciliary dyskinesia (PCD) is a rare autosomal recessive condition often presenting with chronic respiratory infections in early life. Transmission electron microscopy (TEM) is used to detect ciliary ultrastructural defects. In this study, we aimed to assess ciliary ultrastructural defects using quantitative methods on TEM to identify its diagnostic role in confirming PCD. Nasal samples of 67 patients, including 37 females and 30 males (20.3 ± 10.7 years old), with suspected PCD symptoms were examined by TEM. The most common presentations were bronchiectasis: 26 (38.8%), chronic sinusitis: 23 (34.3%), and recurrent lower respiratory infections: 21 (31.3%). Secondary ciliary dyskinesia, including compound cilia (41.4%) and extra-tubules (44.3%), were the most prevalent TEM finding. Twelve patients (17.9%) had hallmark diagnostic criteria for PCD (class 1) consisting of 11 (16.4%) outer and inner dynein arm (ODA and IDA) defects and only one concurrent IDA defect and microtubular disorganization. Also, 11 patients (16.4%) had probable criteria for PCD (class 2), 26 (38.8%) had other defects, and 18 (26.9%) had normal ciliary ultrastructure. Among our suspected PCD patients, the most common ultrastructural ciliary defects were extra-tubules and compound cilia. However, the most prevalent hallmark diagnostic defect confirming PCD was simultaneous defects of IDA and ODA.

## Introduction

**Figure 1:**
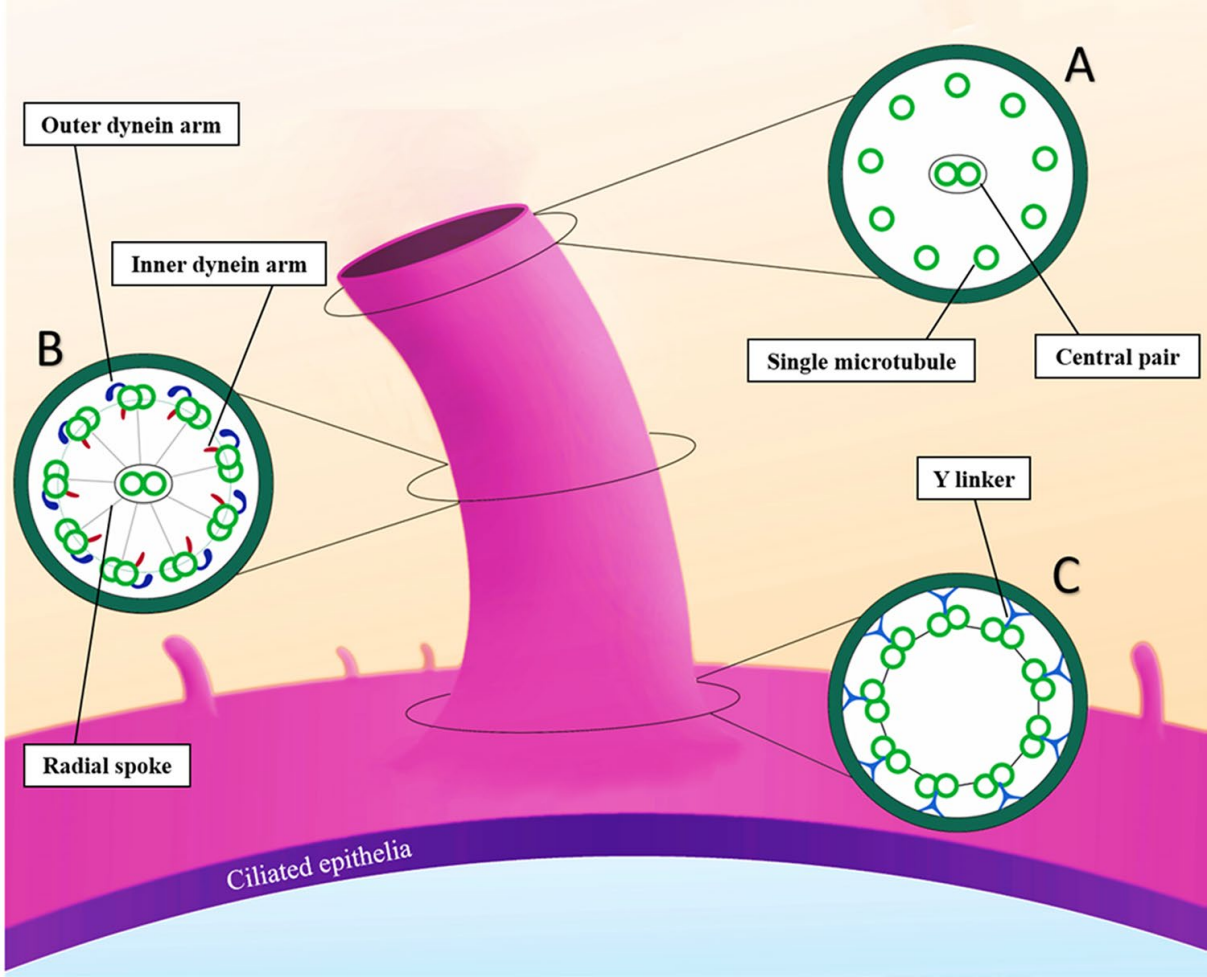
Normal ciliary ultrastructure. (**A**) Tip: cross-sections from the tip of the cilium which consists of single microtubules. It should not be considered a defect. (**B**) Axoneme: it consists of a central pair of microtubules surrounded by nine peripheral microtubule doublets bond to the center pair by supporting radial spokes (known as the 9 + 2 arrangement) Each doublet possesses outer and inner dynein arms. (**C**) Base: cross-section at the base of the cilium, where the central pair is not present. Y-shaped linkers bond microtubule doublets together.

Chronic or recurrent infections of the respiratory tract are common among children. These infections may result from compromised mucociliary clearance. The interaction between ciliary and respiratory mucus is essential in the clearance of the respiratory tract, and any dysfunction in this system results in chronic or recurrent respiratory infections. A clear example is cystic fibrosis (CF)^1,2^. A normal ciliary ultrastructure was firstly introduced by Afzelius, consisting of a central pair of microtubules surrounded by nine peripheral microtubule doublets (known as 9 + 2) bonded to the center by several supporting radial spokes. Each doublet possesses outer and inner dynein arms (ODA and IDA). ODAs are molecular motors, which “walk” along axoneme microtubules and enable bending of the cilium (Fig. 1)^3^. This complex structure is not only found in the upper and lower airways (mucus‐propelling cilia) but is also seen in the middle ears, paranasal sinuses, ependymal lining of the brain ventricles, efferent ducts of the testis, and the oviduct (water‐propelling cilia)^4^.

**Figure 2:**
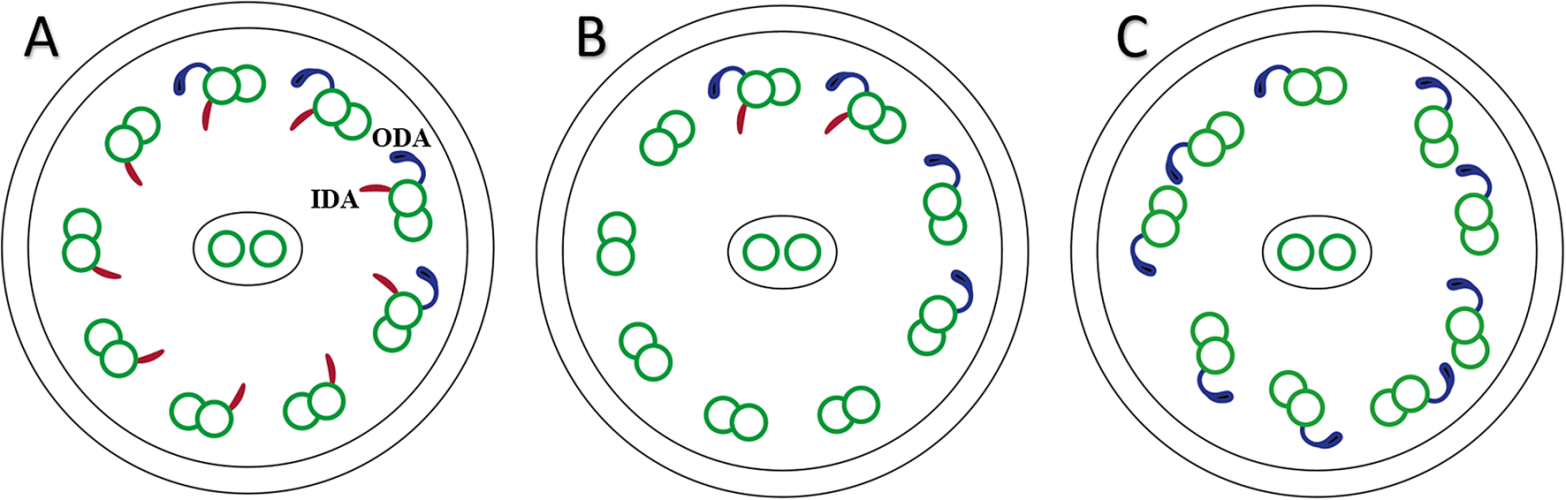
Class 1 (hallmark) defects. (**A**) Outer dynein arm (ODA) defect: the absence of at least 7 out of 9 arms like this schematic cross-section in more than 50% of cilia. (**B**) ODA defect with inner dynein arm (IDA) defect which is defined as the absence of at least 5 out of 9 arms like here in more than 50% of cilia. (**C**) Microtubular disorganization (MTD) and IDA defect: the presence of IDA defect with disruption of 9 + 2 symmetry in more than 25% of cilia.

The ultrastructural ciliary impairments include the defect of the outer and/or inner dynein arms, the microtubular disorganization, and the rare absence of the central pair of microtubules (Fig. 2)^5^. These defects could be a hereditary defect called primary ciliary dyskinesia (PCD) or a post-infectious defect called secondary ciliary dyskinesia (SCD)^6,7^. On-time diagnosis of ultrastructural ciliary defects plays a key role in making clinical decisions and preventing harmful results such as hearing loss and speech impairment caused by recurrent otitis media and irreversible lung damage like bronchiectasis as a result of chronic pulmonary infections^8^.

PCD is an inherited genetic disorder that results from impaired ciliary beating caused mainly by an abnormal ciliary ultrastructure such as the ODA defect with or without the IDA defect^4^. These cilia also play a pivotal role during the embryonic period by determining the laterality of organs; therefore, Situs Inversus is a common finding among PCD patients. PCD cases develop into chronic upper and lower respiratory infections (such as recurrent otitis media, hearing impairment, and recurrent sinusitis), often starting in the early years of life, followed by inevitable adult bronchiectasis^9^. PCD may also manifest as male infertility, as cilia are also present in other organs^4,6^. The prevalence of PCD is estimated to be 1 in 10,000–20,000 newborns and is also more prevalent among consanguineous married family clusters^4,6^. Due to the limited epidemiological data and phenotypic heterogeneity, PCD is rather an unfamiliar disease for most general practitioners. Therefore, these patients are mostly visited more than 50 times without having a definite diagnosis^10^. The lack of accurate and sensitive diagnostic methods has also contributed to the delayed and often incorrect diagnosis of PCD^6^.

This cross-sectional study aimed to assess ciliary ultrastructural defects in terms of demographic and clinical characteristics using quantitative methods on transmission electron microscopy (TEM) to assess the findings of 87 suspected patients. This survey can contribute to a more comprehensive look at the role of EM in evaluating these patients and help improve diagnostic algorithms in PCD diagnosis.

## Results

**Figure 3:**
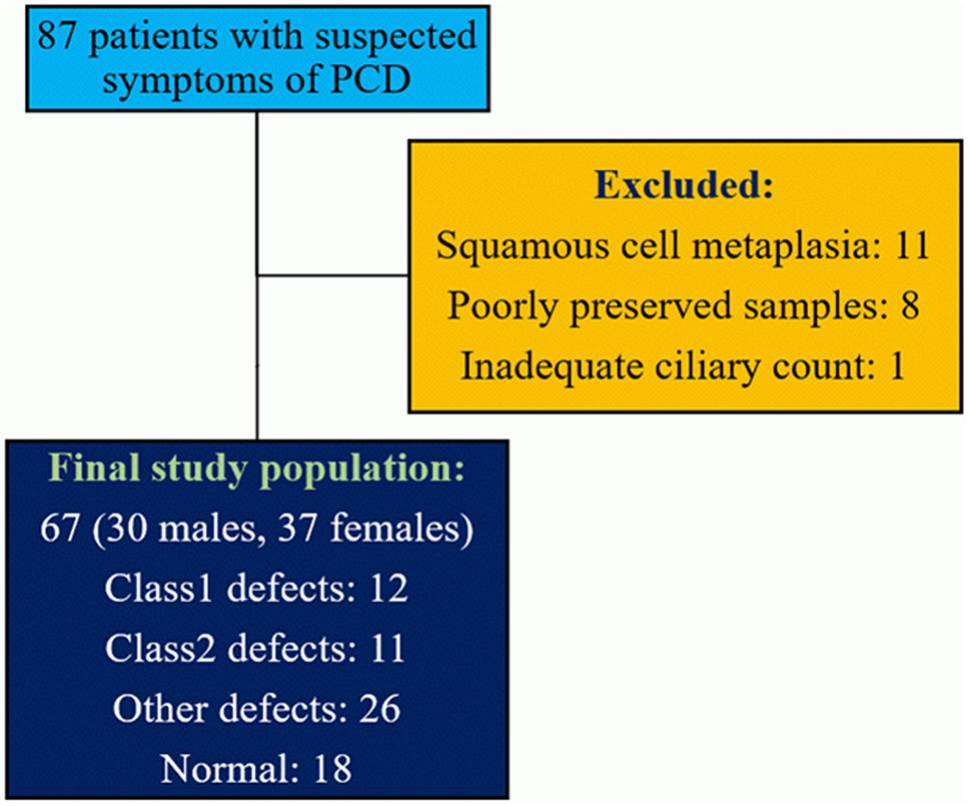
Inclusion and exclusion criteria.

Initially, 87 participants were enrolled in the study. Of the samples, 11 had metaplasia of squamous cells, eight were poorly preserved, and one did not have adequate cilia for examination. Therefore, 20 patients were excluded, and the final study population consisted of 67 participants (Fig. 3).

**Figure 4:**
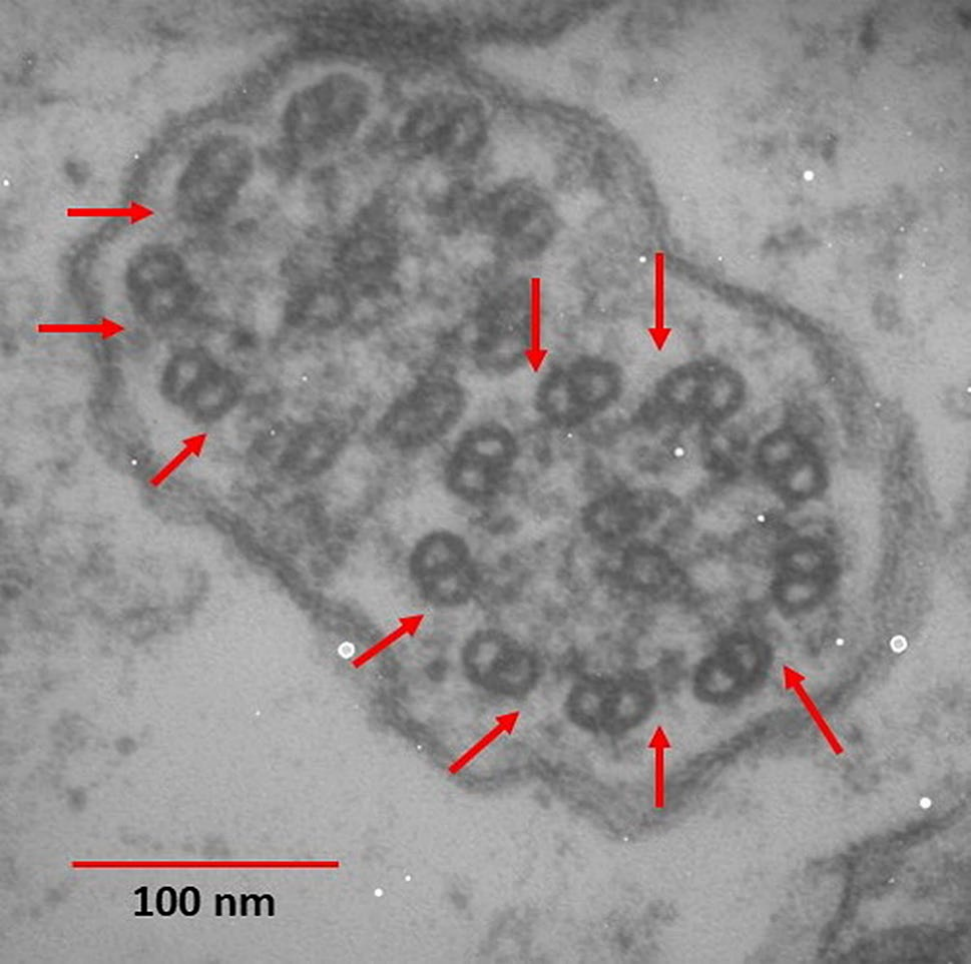
Compound cilia as an SCD. It should be mentioned that some primary defects like ODA defects are also evident in this picture (arrows). (Original magnification: ×140,000). *ODA* outer dynein arm.

Among the patients, 30 (42.9%) were male, and 37 (52.9%) were female. They were between 4 and 47 years of age (mean age = 20.3 ± 10.7 years). Recurrent lower respiratory infections were seen in 21 (31.3%) of the patients. Also, 26 (38.8%) and 23 (34.3%) of the cases had bronchiectasis and recurrent sinusitis, respectively. Infertility was reported in two cases, and nasal polyposis was reported in only one case. Based on TEM analysis, our samples consisted of 12 (17%) hallmark defects, 11 (15.7%) class 2 defects, 26 (37.1%) other defects, and 18 (25.7%) normal TEM findings. Moreover, SCD was also seen: 31 (44.3%) of the patients had extra-tubule, and 29 (41.4%) of them had compound cilia (Fig. 4).

The demographic characteristics and ciliary ultrastructural defects of the cases are shown in Table 1.

**Table 1:** Demographic data, clinical presentation, and ultrastructural ciliary findings of study groups.

	Hallmark defects (n = 12)	Class 2 defects (n = 11)	Other defects (n = 26)	Normal (n = 18)	Total (n = 67)
**Demographic data**					
Mean age	18.6 ± 8.8	23.5 ± 12.3	20.9 ± 11.1	18.7 ± 10.8	20.3 ± 10.7
Gender					
Male	6 (50%)	2 (18%)	14 (54%)	8 (44%)	30 (45%)
Female	6 (50%)	9 (81%)	12 (46%)	10 (56%)	37 (55%)
**Clinical presentations**					
Productive cough	0	0	1 (4%)	1 (5%)	2 (3%)
Bronchiectasis	5 (42%)	5 (45%)	7 (27%)	9 (50%)	26 (39%)
Recurrent sinusitis	3 (25%)	5 (45%)	10 (38%)	5 (28%)	23 (34%)
Infertility	2 (17%)	0	0	0	2 (3%)
Polyposis	0	0	1 (4%)	0	1 (1%)
Recurrent lower respiratory tract infection	6 (50%)	4 (36%)	6 (23%)	5 (28%)	21 (31%)
Auditory symptoms	2 (17%)	2 (18%)	1 (4%)	0	5 (7%)
Situs Inversus	3 (25%)	0	0	0	3 (4%)
**Ultrastructural ciliary findings**					
ODA defect					
ODA absence < 25%	0	0	1 (4%)	0	1 (1%)
ODA absence from 25–50%	0	3 (27%)	0	0	3 (4%)
ODA defect (absence > 50%)	0	0	0	0	0
IDA defect					
IDA absence < 25%	0	2 (18%)	2 (8%)	0	4 (6%)
IDA absence 25–50%	0	0	2 (8%)	0	2 (3%)
IDA defect (absence > 50%)	1 (8%)	3 (27%)	6 (23%)	0	10 (15%)
Both ODA & IDA defects					
ODA & IDA absence < 25%	0	1 (9%)	2 (8%)	0	3 (4%)
ODA & IDA absence 25–50%	0	8 (73%)	0	0	8 (12%)
ODA + IDA defect (absence > 50%)	11 (92%)	0	0	0	11 (16%)
Microtubular disorganization					
Disruption of 9 + 2 symmetry (microtubular disorganization (≤ 25%))	3 (25%)	6 (54%)	14 (54%)	0	23 (34%)
Microtubular disorganization (> 25%)	1 (8%)	0	1 (4%)	0	2 (3%)
Central pair missing					
One central MT missing (≤ 20%)	2 (17%)	0	2 (8%)	0	4 (6%)
One central MT missing (> 20%)	0	0	0	0	0
Both central pair MTs missing (≤ 20%)	3 (25%)	3 (27%)	9 (35%)	0	15 (22%)
Both central pair MTs missing (> 20%)	0	0	0	0	0

*ODA* outer dynein arm, *IDA* inner dynein arm, *MT* microtubule.

Situs Inversus was only seen in the hallmark defects group (three patients), and auditory symptoms were reported mostly in the hallmark defects and class 2 groups (four of the five cases presenting with these symptoms).

Table 2 shows the clinical presentations of the cases with a definite diagnosis of PCD and their ciliary ultrastructural defects detected by TEM.

**Table 2:** Clinical presentations and TEM findings among confirmed cases of PCD.

Case No.	Age	Gender	Clinical presentations						TEM ultrastructure				
			RRI	AS	RS	SI	B	I	Class 1		Others		
									ODA + IDA	IDA + MTD	Extra-tubule	Compound tubule	Both of the central pair missing
29	14	M	+	−	−	−	−	−	+	−	+	+	−
49	17	F	+	−	−	−	−	−	+	−	+	−	−
70	10	F	+	+	−	−	−	−	+	−	+	+	−
32	13	M	+	−	−	−	−	−	+	−	−	−	−
33	19	M	−	−	+	+	−	−	+	−	−	−	−
50	4	F	+	−	−	−	−	−	+	−	−	−	−
58	24	F	−	−	+	−	+	+	+	−	+	−	−
65	18	F	−	−	−	−	+	−	+	−	−	−	−
46	32	M	−	−	+	+	+	+	+	−	+	−	
82	24	F	−	−	−	−	+	−	+	−	−	+	
86	34	M	+	−	−	+	−	−	−	+	+	+	+
87	15	M	−	+	−	−	+	−	+	−	−	+	

*PCD* primary ciliary dyskinesia, *TEM* transmission electron microscopy, *M* male, *F* female, *RRI* recurrent lower respiratory infection, *AS* auditory symptoms, *RS* recurrent sinusitis, *SI* Situs Inversus, *B* bronchiectasis, *I* infertility, *ODA* outer dynein arm, *IDA* inner dynein arm, *MTD* microtubular disorganization.

**Figure 5:**
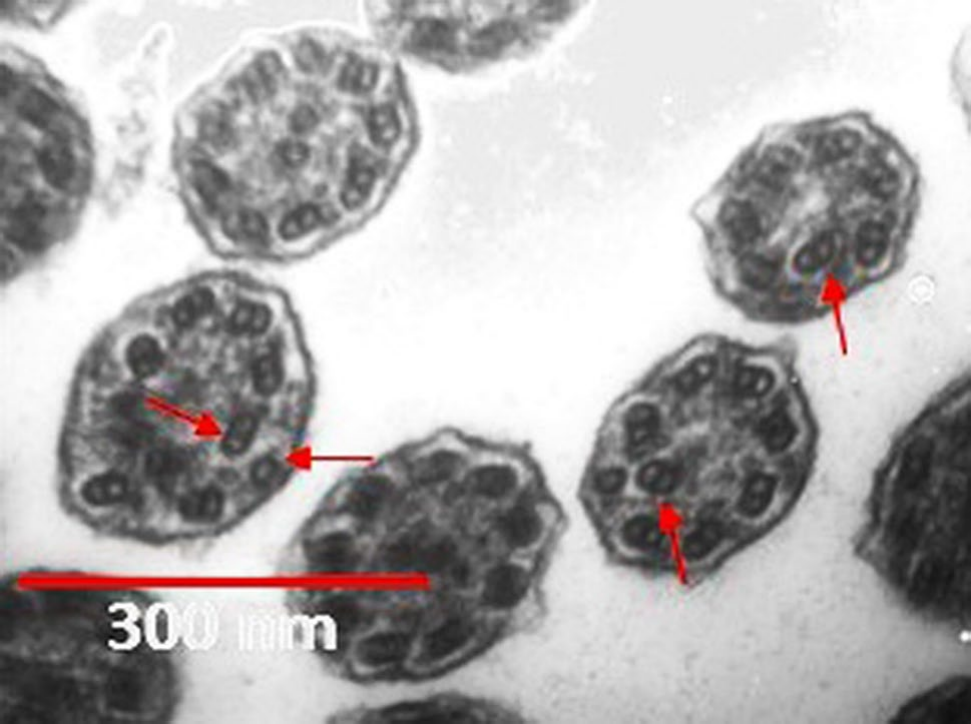
Microtubular disorganization (arrows) (Original magnification: ×50,000).

**Figure 6:**
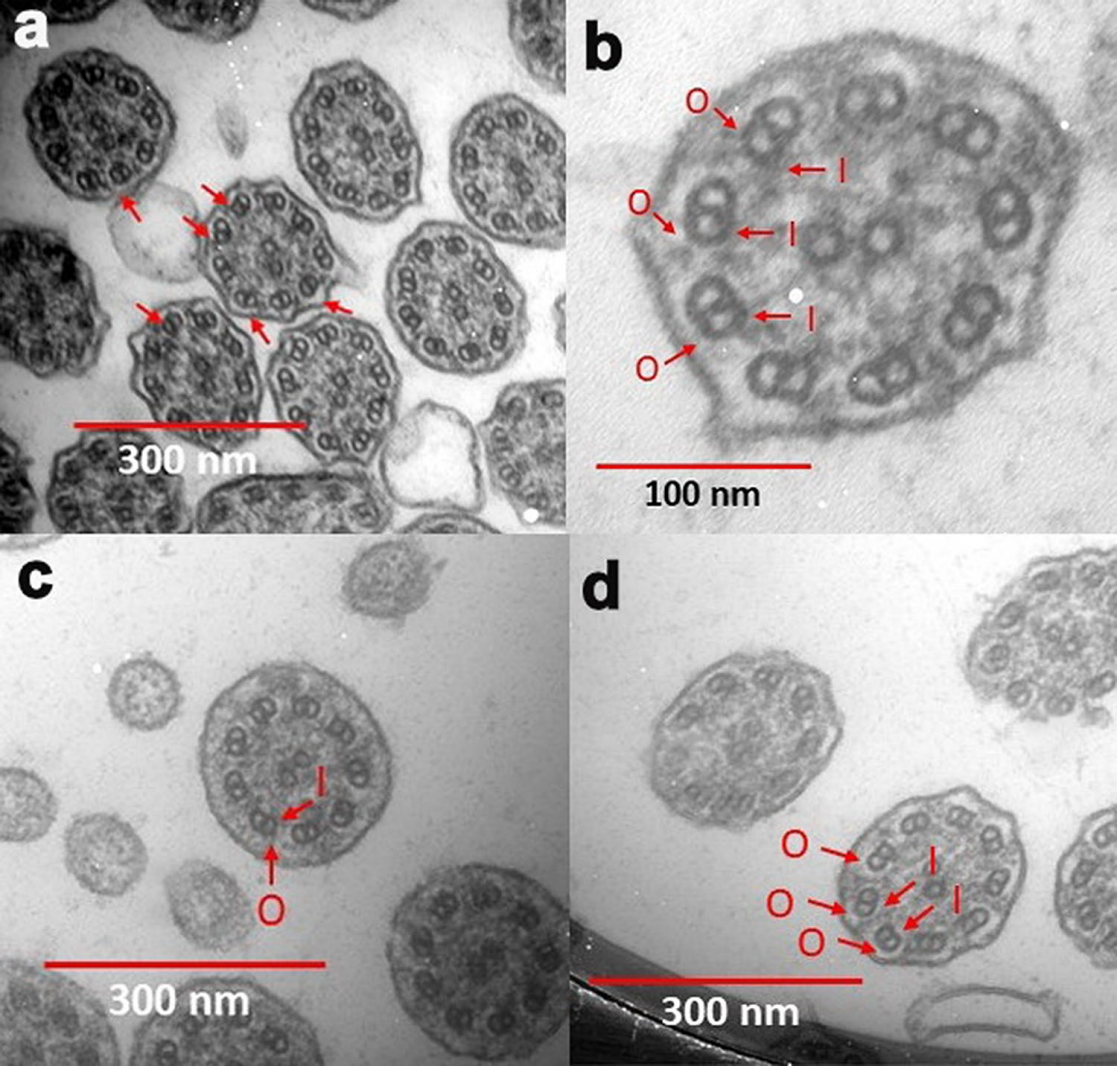
IDA and ODA defects. (**a**) ODA defects (arrows). (**b**, **c**, **d**) Some absence of IDA (I) and ODA (O) are marked. (Original magnification: **a**, **c** & **d**: ×50,000, **b**: ×140,000).

Figure 5 shows some examples of microtubular disorganization and Fig. 6 shows some examples of IDA and ODA defects in our patients.

## Discussion

This survey was the first study conducted in Iran to evaluate ciliary ultrastructural defects among patients presenting with symptoms attributable to PCD. In the current study, 12 patients had a definite diagnosis of PCD. Eleven patients were indicated as PCD cases, as they had class 2 defects with compatible symptoms and other diagnoses were ruled out. Therefore, they benefited from the early diagnosis of PCDs, which is important because the earlier onset of clinical intervention prevents permanent bronchiectasis and other insidious lung tissue damages^11^. We also reported 18 cases with normal patterns in TEM. However, 30% of the PCD patients might present normal TEM findings, such as those with large dynein heavy chain *Dynein Axonemal Heavy Chain 11* (*DNAH11*) and defects in *HYDIN*^12^.

According to the European Respiratory Society guidelines for the diagnosis of primary ciliary dyskinesia, it is highly recommended to screen patients with recurrent productive cough, chronic rhinitis, persistent middle ear disease, congenital heart disease, and neonatal respiratory infections for PCD. Also, PCD patients' siblings should undergo PCD investigations^13,14^. In our cases, the most prominent symptoms among the patients were bronchiectasis (39%) and persistent sinusitis (34%). However, recurrent lower respiratory tract infections were even more common in the hallmark defects group than in all subjects (50% compared to 31%). It is pivotal to note that five patients in the hallmark defects group had bronchiectasis; however, they did not report productive cough. The probable reason for this discrepancy can be the strict criteria used for productive cough's definition in this study. These patients might suffer from ongoing non-productive coughing or ineffective coughing as a result of a defect in mucociliary clearance^15^*.*

PCD and SCD may have similar symptoms. However, in PCD, TEM findings are permanent and seen in most cilia. Secondary changes, however, could be the result of former infections and would recover over time or by cell culture^7,16,17^. In our study, the most common ultrastructural ciliary defects were extra-tubules (44.3%) and compound cilia (41.4%), and examples of SCD were identified in almost half of the sample population (Fig. 4).

Furthermore, changes such as microtubular disorganization and central pair abnormalities may be common between PCD and SCD. Therefore, to confirm PCD diagnosis, other specific diagnostic methods should be used. Repeating the biopsy and considering a sufficient healthy interval before re-sampling also help to differentiate between PCD and SCD more precisely^12^. Almost 30% of our samples had IDA absence, overlapping between PCD and non-PCD cases; the probable reason may be that it is difficult to see IDA by TEM^18–20^. Therefore, it is highly suggested to repeat the biopsy, especially for this group^21^. Besides, in the event of an isolated IDA defect, the cultivation may result in a reversal and can exclude the diagnosis of PCD^7^. To avoid misinterpretation as an ultrastructural defect, great attention should be paid to the difference between the normal shape of the tip and the base of the cilia and its body, because the diameter of the cilium varies along its length, it is wider at the base and narrower at the tip^12^ (Figs. 1 and S1).

**Figure 7:**
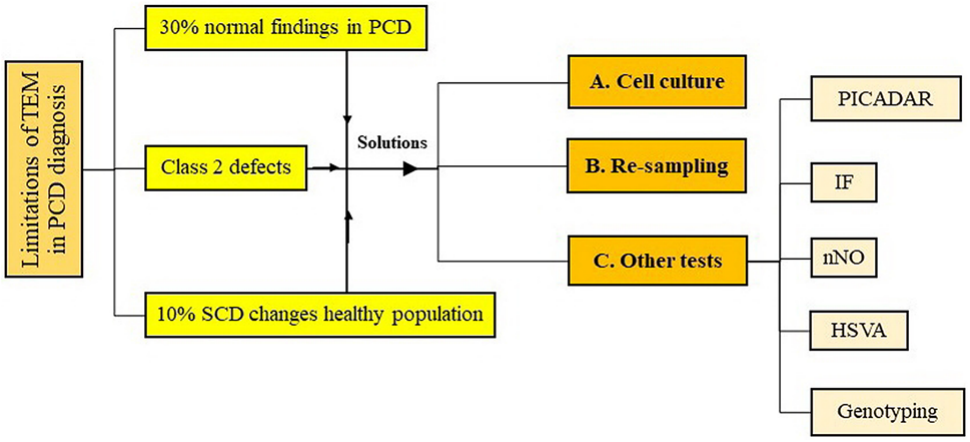
Challenges of PCD diagnosis and possible solutions. *TEM* transmission electron microscopy, *PCD* primary ciliary dyskinesia, *SCD* secondary ciliary dyskinesia, *PICADAR* PrImary CiliAry DyskinesiA Rule, *IF* immunofluorescence, *nNO* nasal nitric oxide, *HSVA* high-speed video analysis.

To date, PCD diagnosis has remained a significant medical challenge in resource-limited regions, and a variety of diagnostic approaches are used in different countries based on their local guidelines. Saccharine testing was once used for PCD screening but is no longer considered a reliable method^22^. Although TEM was previously thought to be a gold-standard test, there is currently no stand-alone diagnostic test for PCD, and a combination of several techniques is required to confirm the disease^23^. To date, nasal nitric oxide (nNO) and high-speed video microscopy analysis (HSVA) have been used in combination with TEM to confirm a PCD diagnosis based on local diagnostic algorithms. However, the European Respiratory Society guideline^13^ labels nNO for patients under the age of 6 years and HSVA as a "weak recommendation" and still strongly advocates TEM. The limitation of TEM in PCD diagnosis, particularly in class 2 defects and SCD changes, prompts researchers to use other diagnostic tests or repeat sampling to reach the definite diagnosis (Fig. 7).

Reviewing the literature^13,22,23^ and considering the cost of TEM analysis, we think nNO in combination with the primary ciliary dyskinesia rule (PICADAR) score^24^ is a proper initial screening test for an adult. However, further studies are needed in this regard. From our experience and due to its high specificity, TEM is a reliable tool for diagnosing PCD. However, up to 30% of PCD patients have normal TEM results^12^. Furthermore, there is no confirmed diagnosis for patients with class 2 defects by using TEM alone. Therefore, we suggest performing other tests for these groups of patients, along with re-sampling or cell culture, to make further decisions^12^.

These patients would have a regular joyful life only if diagnosed on time and given proper antibiotic treatment for each respiratory infection. Regular physiotherapy is helpful, as well^25^. On the other hand, recurrent infections will result in respiratory complications, including recurrent pneumonia, bronchiectasis, parenchymal fibrosis, and hypoxemia induced by ventilation/perfusion mismatch. If hypoxemia persists for a long time, it may lead to right ventricular failure. Hence, early diagnosis would prevent irreversible consequent complications occurred during the clinical course and reduce the burden of disease and its great cost for the health system^26^.

Our work has led us to conclude that the most prevalent hallmark diagnostic defect among suspected PCD patients was simultaneous lack of IDA and ODA. Further studies with other PCD diagnostic methods and their comparison with TEM are needed to establish a local guideline in western Asian countries.

## Methods

### Study design and patient enrollment

This prospective cohort study was conducted on referral patients from several affiliated centers to the TEM of the central laboratory, School of Medicine, the Shahid Beheshti University of Medical Sciences, Tehran, Iran, from 2017 to 2019. The patients had at least one of the suspected symptoms of PCD listed in Table S1 or positive history of PCD in the family (particularly in siblings and consanguineous marriages).

The patients were previously evaluated for probable differential diagnoses, including asthma (using both salbutamol and methacholine challenge tests), cystic fibrosis, immunological disorders, and chronic gastroesophageal reflux (depending on their past medical history). If one of the above diagnoses was confirmed and the patient's symptoms were justified with this diagnosis, these patients would be excluded.

Overall, 87 patients were enrolled in the study and tested for ciliary ultrastructural defects. Informed consent was taken from the participants or their parents in the case of children. The study was approved by the Ethics Committee of the Medical School, the Shahid Beheshti University of Medical Sciences (Code = IR.SBMU.MSP.REC.1397.382). All methods were carried out following relevant guidelines and regulations.

All the participants filled out a questionnaire designed by the expert medical team. The questionnaire provided demographic data (age and sex) and their clinical presentations, including chronic productive cough, recurrent sinusitis, recurrent lower respiratory infections, auditory symptoms, Situs Inversus, and infertility in both genders. The study defined the chronic productive cough as a purulent cough lasting more than 8 weeks^15^. Also, recurrent sinusitis was defined as four or more episodes of sinusitis with symptomless intervals each year^27^ and chronic sinusitis as a condition in which the symptoms last for more than 12 weeks^28^; we considered both of these definitions. Auditory symptoms included recurrent, chronic otitis and hearing loss. Infertility was defined based on past medical history.

### TEM analysis

An expert otolaryngologist collected ciliary respiratory mucosa. It is important to note that sampling was avoided in patients with upper or lower respiratory tract infections, and they were asked to return without any respiratory symptoms after 6 weeks. Nasal samples were fixed by glutaraldehyde 2.5%, and then the second fixation was performed using osmium tetroxide 1% in 0.1 M veronal acetate buffer. The samples were dehydrated by passing through the ethanol series and then embedded in the epoxy-containing resin. The ultrathin sections of lead citrate and uranyl acetate were stained to provide an appropriate ultrastructural view. These sections were evaluated using a ZEISS EM900 transmission electron microscope to detect ciliary ultrastructural defects. At least 50 intact cilia from healthy areas with minimal artifacts were examined for each case^12^.

In addition, poorly preserved samples, samples with squamous cell metaplasia resulting in non-detectable cilia in the light-microscope or samples with an insufficient number of cilia (less than 50 sections for each patient) were excluded (Fig. 3).

Ultrastructural defects are classified into two main classes, according to the International Consensus guideline for reporting transmission electron microscopy results in the diagnosis of PCD^12^.

Class 1 defects include:

1. ODA defects: It is defined as the absence of more than seven out of nine arms in each cilium that must be seen in more than 50% of cilia.
2. Both ODA and IDA defects: ODA defects with IDA defects are defined as the absence of more than five out of nine arms in each cilium that must be seen in more than 50% of cilia (Fig. S2).
3. Microtubular disorganization and IDA defects: Microtubular disorganization is defined as the disruption of 9 + 2 symmetry in more than 25% of cilia (Fig. 5).

Class 2 defects include:

1. Central complex defect: It is defined as the absence of one or two central microtubules in more than 20% of examined cilia or lateral transposition of central pair. Also, abnormal counts like 8 + 1 are in this category (Fig. S3).
2. Mislocalization of basal bodies with few or no cilia: It refers to very low count of cilia along with basal bodies failure to dock the apical surface of each cilium.
3. Microtubular disorganization with IDA present: In this criterion, the disruption of 9 + 2 symmetry is seen in less than 25% of cilia.
4. ODA absence from 25–50% of cilia cross-sections
5. Combined IDA and ODA absence from 25–50% of cilia cross-sections

Therefore, based on these findings, the TEM findings of the patients were classified into four groups (Fig. 3) (Table S2).

1. Hallmark defects defined as the existence of at least one of the class 1 defects
2. Class 2 defects defined as the existence of at least one of the class 2 defects with other supporting evidence
3. Other defects: having other ciliary ultrastructural defects that are not classified in class 1 or 2 defects.
4. Normal: normal ciliary ultrastructure

### Statistical analysis

The data were recorded in Microsoft Excel 2019 (Supplementary file 2) and then imported to the SPSS version 21 (the URL of SPSS version 21 was https://www.ibm.com/support/pages/spss-statistics-210-available-download). Data analysis was conducted using descriptive analysis of each patient's demographic and ciliary ultrastructural characteristics after splitting them into hallmark defects, class 2 defects, other defects, and normal groups.

## Supplementary Information

Supplementary Information 1.

Supplementary Information 2.

## Data Availability

The materials and data of this study are available from the corresponding author and can be provided upon reasonable request.
