# Supplementary material for: Transmission electron microscopy study of suspected primary ciliary dyskinesia patients
Source: Sci Rep. 2022 Feb 11;12:2375. doi: 10.1038/s41598-022-06370-w (PMC8837606; doi:10.1038/s41598-022-06370-w)
Supplement: Supplementary file 1 — Supplementary Information 1. [file 41598_2022_6370_MOESM1_ESM.docx]

**Transmission Electron Microscopy Study of Suspected Primary Ciliary Dyskinesia Patients**

[Mitra Rezaei](https://onlinelibrary.wiley.com/action/doSearch?ContribAuthorStored=Rezaei%2C+Mitrasadat)^1,2^, Amirali Soheili^3^, [Seyed Ali Ziai](https://onlinelibrary.wiley.com/action/doSearch?ContribAuthorStored=Ziai%2C+Seyed+Ali)^4^, [Atefeh Fakharian](https://www.ncbi.nlm.nih.gov/pubmed/?term=Fakharian%20A%5BAuthor%5D&cauthor=true&cauthor_uid=21572699)^5^, Hossein Toreyhi^3^, [Mihan Porabdollah](https://pubmed.ncbi.nlm.nih.gov/?term=Porabdollah+M&cauthor_id=33262795)^5^, Jahangir Ghorbani^5^, Mahboobeh Karimi-Galougahi^6^, [Seyed Alireza Mahdaviani](https://pubmed.ncbi.nlm.nih.gov/?term=Mahdaviani+SA&cauthor_id=29512373)^7^, Maryam Hasanzad^7^, [Alireza Eslaminejad](https://pubmed.ncbi.nlm.nih.gov/?term=Eslaminejad+A&cauthor_id=33262795)^6^, Hossein Ali ghaffaripour^7^, Saied Mahmoudian^5^, Zahra Rodafshani^8^, [Maryam Sadat Mirenayat](https://pubmed.ncbi.nlm.nih.gov/?term=Mirenayat+MS&cauthor_id=33262795)^5^, [Mohammad Varahram](https://pubmed.ncbi.nlm.nih.gov/?term=Varahram+M&cauthor_id=33631512)^5^, [Majid Marjani](https://pubmed.ncbi.nlm.nih.gov/?term=Marjani+M&cauthor_id=33631512)^2*^, [Payam Tabarsi](https://pubmed.ncbi.nlm.nih.gov/?term=Tabarsi+P&cauthor_id=33631512)^2^, [Davood Mansouri](https://pubmed.ncbi.nlm.nih.gov/?term=Mansouri+D&cauthor_id=33498051)^2^, [Hamid Reza Jamaati](javascript:;)^5^, [Ali Akbar Velayati](https://pubmed.ncbi.nlm.nih.gov/?term=Velayati+AA&cauthor_id=33262795)^2^

^1^Department of Pathology, School of Medicine, Shahid Beheshti University of Medical Sciences, Tehran, Iran

^2^Clinical Tuberculosis and Epidemiology Research Centre, National Research Institute of Tuberculosis and Lung Diseases (NRITLD), Shahid Beheshti University of Medical Sciences, Tehran, Iran

^3^Medical student research committee, School of Medicine, Shahid Beheshti University of Medical Sciences, Tehran, Iran

^4^Department of Pharmacology, School of Medicine, Shahid Beheshti University of Medical Sciences, Tehran, Iran

^5^Chronic Respiratory Diseases Research Center (CRDRC), National Research Institute of Tuberculosis and Lung Diseases (NRITLD), Shahid Beheshti University of Medical Sciences, Tehran, Iran

^6^Tracheal Diseases Research Center, National Research Institute of Tuberculosis and Lung Diseases, Shahid Beheshti University of Medical Sciences, Tehran, Iran.

^7^Pediatric Respiratory Disease Research Centre, National Research Institute of Tuberculosis and Lung Diseases (NRITLD), Shahid Beheshti University of Medical Sciences, Tehran, Iran

^8^Central Lab, School of Medicine, Shahid Beheshti University of Medical Sciences, Tehran, Iran

*Correspondence: Dr. [Majid](https://onlinelibrary.wiley.com/action/doSearch?ContribAuthorStored=Rezaei%2C+Mitrasadat) Marjani, Clinical Tuberculosis and Epidemiology Research Centre, National Research Institute of Tuberculosis and Lung Diseases (NRITLD), Shahid Beheshti University of Medical Sciences, Tehran, Iran, [Orcid ID:](http://orcid.org/0000-0002-1102-8119) 0000-0003-1466-8645 E-mail: [marjani@sbmu.ac.ir](mailto:marjani@sbmu.ac.ir); Telefax: +98 2126109590

**Keywords:** ciliary dyskinesia, dynein arm, transmission electron microscopy, ciliary ultrastructure

**Supplementary figures and tables:**

**
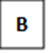

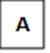
**
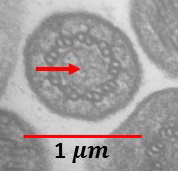

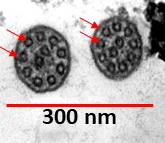


**Figure S1** A) Sections from the base of cilium, central pair is not present (arrow) (Original magnification: ×12,000) and B) tip of cilium consisted of single microtubules (arrows) (original magnification: ×50,000) of cilia which should not be misdiagnosed as a defect.

**Table S1:** Suspected presentations of PCD

| 1. Respiratory distress in a newborn |
| --- |
| 1. Situs Inversus, heterotaxy, poly- or asplenia |
| 1. Chronic otitis media |
| 1. Hearing loss |
| 1. Neonatal pneumonia |
| 1. Chronic nasal congestion and mucopurulent rhinitis |
| 1. Nasal polyposis |
| 1. Chronic pan-sinusitis |
| 1. Chronic productive cough |
| 1. Recurrent pneumonia |
| 1. Bronchiectasis of unknown etiology |
| 1. Airway obstruction, gas trapping, or treatment-resistant asthma |
| 1. Unexplained male and female infertility |
| 1. Hydrocephalus |
| 1. Retinitis pigmentosa |


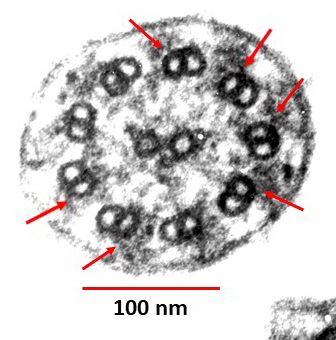


**Figure S2:** Outer and inner dynein arm defect. Absence of the full ODA and IDA structures from the majority of microtubular doublets. (arrows)

**Abbreviations:** ODA: outer dynein arm; IDA: inner dynein arm.

**
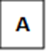

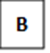

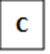
**


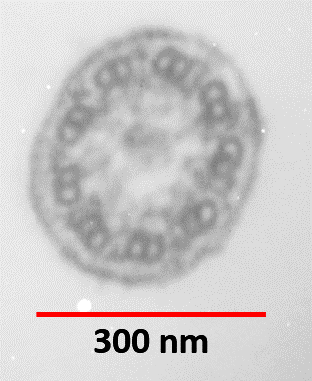

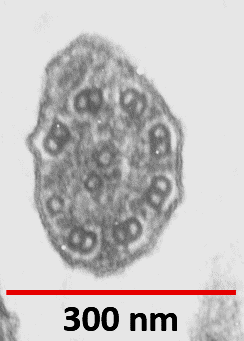

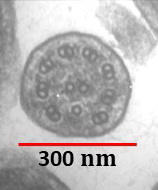


**Figure S3:** Variety of central complex defects. A) absence of both central microtubules (9+0), B) The transposition of an outer couple into the center of cilium as a result of one of the central pair missing (9+1), C) abnormal counts of microtubules (8+1). (Original magnifications: ×85,000).

**Table S2:** Summarized of common morphologic abnormalities in PCD in comparison to normal cilium

| 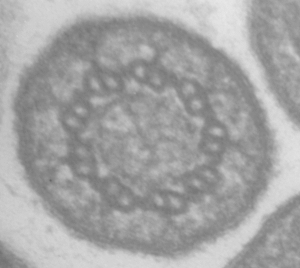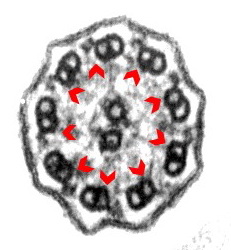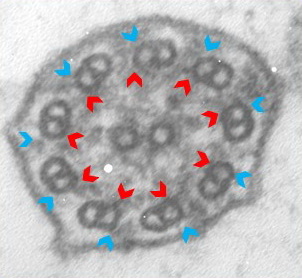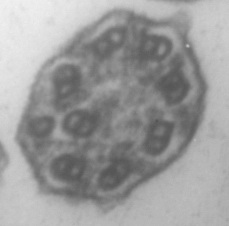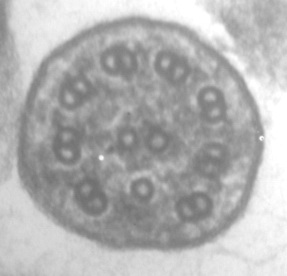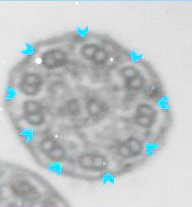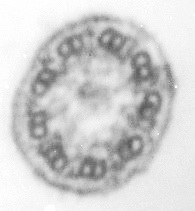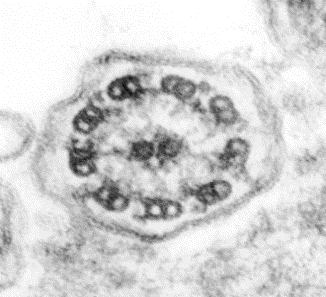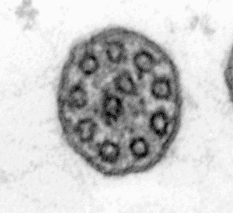 |  |  |
| --- | --- | --- |
| Base of normal cilium | Axoneme of normal cilium | Tip of normal cilium |
|  |  |  |
| Absent ODA | Absent IDA | Absent of both IDA & ODA |
|  |  |  |
| Microtubular disorganization | 9+1 transposition defect | Absent central pair |

Blue arrowhead: absent ODA; Red arrowhead: absent IDA

**Abbreviations:** PCD: primary ciliary disorder; ODA: outer dynein arm; IDA: inner dynein arm
